# Real-world outcomes of different treatment strategies in patients with diabetes and three-vessel coronary disease: a mean follow-up 6.3 years study from China

**DOI:** 10.1186/s12933-020-01193-3

**Published:** 2021-01-11

**Authors:** Xueyan Zhao, Lianjun Xu, Lin Jiang, Jian Tian, Yin Zhang, Dong Wang, Kai Sun, Bo Xu, Wei Zhao, Rutai Hui, Runlin Gao, Lei Song, Jinqing Yuan

**Affiliations:** State Key Laboratory of Cardiovascular Disease, Fuwai Hospital, National Center for Cardiovascular Diseases, Chinese Academy of Medical Sciences and Peking Union Medical College, No. 167 Beilishi Road, Xicheng District, Beijing, China

**Keywords:** Triple-vessel coronary disease, Diabetes, Treatment strategies, Prognosis

## Abstract

**Background:**

Patients with diabetes and triple-vessel disease (TVD) are associated with a high risk of events. The choice of treatment strategies remains a subject of discussion. In the real-world, we aim to compare the outcomes of medical therapy (MT), coronary artery bypass grafting (CABG), and percutaneous coronary intervention (PCI) treatment strategies in patients with diabetes and TVD.

**Methods:**

A total of 3117 consecutive patients with diabetes and TVD were enrolled. The primary endpoint was all-cause death and the secondary endpoint was major adverse cardiac and cerebrovascular events (MACCE, composite of all-cause death, myocardial infarction, or stroke).

**Results:**

During the mean follow-up of 6.3 ± 2.6 years, 573 (18.4%) deaths and 1094 (35.1%) MACCE occurred. Multivariate analysis showed that PCI (hazard ratio [HR] 0.40, 95% confidence interval [CI] 0.32–0.51) and CABG (HR 0.33, 95% CI 0.26–0.44) were associated with a lower risk of death compared with MT, with no difference between the PCI and CABG groups. When MACCE was the endpoint, PCI (HR 0.71, 95% CI 0.60–0.84) and CABG (HR 0.48, 95% CI 0.39–0.57) had a lower risk than MT. CABG was associated with a significantly lower risk of MACCE compared with PCI (HR 0.67, 95% CI 0.55–0.81), which was mainly attributed a lower risk in myocardial infarction, but a higher risk of stroke.

**Conclusions:**

In this big real-world data and intermediate-term follow-up study, for patients with diabetes and TVD, PCI and CABG were associated with a lower risk of death and MACCE more than MT. The results suggest the importance of appropriate revascularization for diabetic patients with TVD. However, CABG was not associated with a lower risk of death, but with a lower risk of MACCE, compared with PCI. In the future, we perhaps should strengthen comprehensive treatment in addition to PCI or CABG.

## Background

Patients with multi-vessel coronary artery disease (MVD) and diabetes mellitus often have diffuse atherosclerosis and high risk of cardiovascular events [1, 2]. The choice between percutaneous coronary intervention (PCI) and coronary artery bypass grafting (CABG) in diabetic patients with triple-vessel coronary artery disease (TVD) remains a subject of intense discussion and debate. Most previous studies have shown that CABG reduces the risk of major adverse cardiac and cerebrovascular events (MACCE) in patients with diabetes and MVD compared with PCI [3–5]; however, the results of CABG and PCI for all-cause death were not completely consistent [3, 6–10]. A meta-analysis [6] showed that CABG had a mortality benefit over PCI in patients with MVD and diabetes who did not present with acute myocardial infarction (MI). Future Revascularization Evaluation in Patients with Diabetes Mellitus: Optimal Management of Multivessel Disease (FREEDOM) was a landmark study, when followed up for 3.8 years [4], although a higher mortality rate was observed in the PCI group (P = 0.049) than in the CABG, the authors cautiously pointed out that the study was not sufficient for determining all-cause deaths. Among them, 943 patients had a prolonged follow-up (median of 7.5 years), and there was no significant difference in all-cause death between the groups (P = 0.076) [7]. It is worth noting that the results of the recent 10-year Synergy between Percutaneous Coronary Intervention with Taxus and Cardiac Surgery (SYNTAX) study [10] showed no difference in all-cause death between PCI and CABG.

There is no doubt that these randomized controlled trials have many advantages. However, in these studies for diabetes and MVD, the strict exclusion criteria always excluded acute MI, left main disease, heart failure, and recent stroke. In addition, Some patients were not eligible for inclusion in the randomized trials because coronary lesion is too complex to be treated with PCI or surgical risk considered too high for CABG [6]. Although the guideline [11] recommended CABG as the preferred treatment for patients with diabetes and TVD, in the real world, a considerable number of patients with diabetes and TVD receive PCI treatment or even just medical treatment (MT) alone. Therefore, the prognosis of these patients in the real world deserves attention.

Therefore, we aimed to compare the intermediate-term follow-up outcomes of different treatment strategies, including MT, PCI, and CABG, for all-cause death and MACCE in patients with diabetes and TVD in the real world. We further examined whether the SYNTAX score affects the results of different treatment strategies.

## Methods

### Study design and patients

**Fig. 1:**
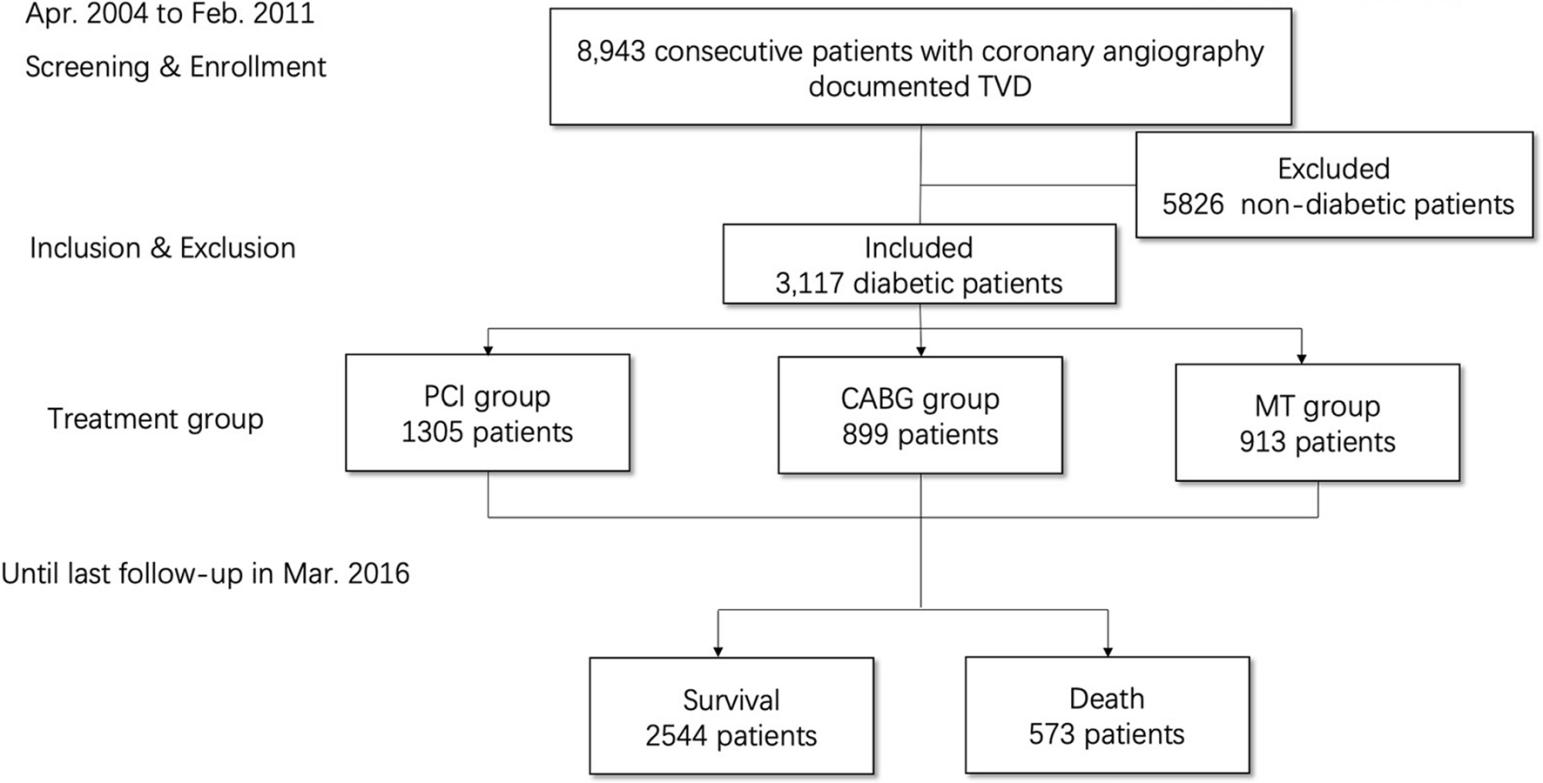
Patient flow chart for the study cohort. Flowchart depicts patients enrolled. *TVD * triple-vessel coronary disease; *PCI* percutaneous coronary intervention; *CABG* coronary artery bypass grafting

This study was a prospective and single-center study. A total of 8943 consecutive patients with TVD were enrolled from April 2004 to February 2011 in Fuwai Hospital (Beijing, China). The methodology was described previously [12]. The inclusion criteria were as follows: patients were diagnosed as having TVD (defined as angiographic stenosis of ≥ 50% in all three main epicardial coronary arteries, with or without left main artery involvement) and were willing to be followed up. There were no pre-specified exclusion criteria. Among them, 3117 patients with diabetes were included in the current analysis (Fig. 1). Diabetes mellitus was defined according to the guidelines for Prevention and Treatment of Diabetes in China. The diagnostic criteria for diabetes were diabetic symptoms with random venous blood glucose levels ≥ 11.1 mmol/L, fasting venous blood glucose levels ≥ 7.0 mmol/L, oral glucose tolerance test 2-hour blood glucose levels ≥ 11.1 mmol/L, or use of antidiabetic drugs. Clinical baseline information, procedural or operative characteristics, and outcome data were collected in detail by independent research personnel. The SYNTAX score [13] was calculated by independent researchers who were blinded to the clinical data using a web calculator (http://www.syntaxscore.com). The study was approved by the ethics committee of Fuwai Hospital. All patients signed informed consents.

### Study procedures

The choice of PCI, CABG, or MT was mainly followed the guidelines and based on clinical and angiographic features, physical condition (comorbidities, malignant tumor, frailty, etc.), complexity, and was discussed by physicians and surgeons, combined with the choice of patients. The PCI strategy and stent type were left to the physician’s discretion. All of the patients undergoing PCI were prescribed aspirin plus clopidogrel. If patients with selective PCI were not taking long-term aspirin and clopidogrel, they received 300 mg aspirin and 300 mg clopidogrel orally at least 24 hours before the procedure. After PCI, patients were prescribed aspirin 100 mg once daily indefinitely, and clopidogrel 75 mg once daily for at least 1 year.

In the CABG group, the left internal mammary artery was routinely used to graft to the left anterior descending artery and completed by venous grafts to other coronary branches with standard bypass techniques. The procedure was performed by surgeons experienced in on-pump or off-pump surgery at the operator’s discretion.

Patients with neither PCI nor CABG treatment were allocated to the MT alone group. Throughout the study period, all patients, regardless of whether they were in the MT group, PCI group, or CABG group, were strongly recommended to use secondary prevention medication according to the clinical guidelines [14, 15], while emphasizing lifestyle changes and medication adherence.

### Endpoints and follow-up

The primary endpoint was all-cause death. Secondary endpoints were MACCE, which were a composite of all-cause death, MI, or stroke. Clinical information of in-hospital outcome was obtained by reviewing medical records. Follow-up was completed by survey via telephone, letter, or hospital visit. All patients had at least one follow-up visit. At the end of the last follow-up in 2016, the corresponding response rate was 87.01% in the diabetic population. All endpoints were carefully adjudicated by a group of independent clinical physicians. Data quality was controlled by various methods, such as training of investigators, blinded filling in of questionnaires, and telephone recording.

### Statistical analysis

All patients who were enrolled in the analysis completed at least one follow-up. Patients who were lost to follow-up were calculated according to censored data. Summary statistics are shown as frequency and percentage for categorical variables and as mean with standard deviation for continuous variables. Continuous variables were compared by one-way ANOVA and categorical variables were compared with the Pearson chi-square test. The cumulative survival rate was calculated by the Kaplan–Meier method, and differences among treatment groups were assessed by the log-rank test. Multivariate Cox regression was used to identify predictors of all-cause death and MACCE. The Cox proportional hazard model was used to estimate the hazard ratios (HRs) and their corresponding confidence intervals (CIs) for the combined endpoint and each individual endpoints. Multivariate analysis was performed by adjusting for age, sex, body mass index, hypertension, hyperlipidemia, chronic obstructive pulmonary disease, peripheral vessel disease, smoking history, previous PCI, prior CABG, high-sensitivity C-reactive protein levels, creatinine clearance rate, left ventricular ejection, and baseline SYNTAX score, and the variables were determined according to univariate results and clinical relevance. The effects of different SYNTAX groups (low risk [≤ 22], medium risk [23–32], and high risk [≥ 33]) on the results were further evaluated. Consistency of treatment effects in different subgroups was assessed by Cox regression models with tests for interaction. All statistical tests were two-sided with a significance level of 0.05. Statistical analysis was performed with SAS 9.4 software (SAS Institute Inc., Chicago, IL).

## Results

### Patients’ characteristics

A total of 3117 patients with diabetes and TVD were included in the study. Among these patients, 1305 (41.9%) were in the PCI group, 899 (28.8%) were in the CABG group, and 913 (29.3%) were in the MT group; 687 (22.04%) were acute myocardial infarction; 1230 (39.46%) were unstable angina pectoris; and 1200 (38.50%) were stable angina pectoris. SYNTAX scores of ≤ 22 were found in 1088 patients, SYNTAX scores of 23–32 were found in 1170 patients and SYNTAX scores of ≥ 33 were found in 767 patients. After PCI, excluding previous CABG 42 patients, residual SYNTAX sore 0 points 67 patients; > 0 and ≤ 8 points 465 patients; > 8 points 731 patients. Comparison between PCI, CABG and MT groups, age, gender, chronic renal insufficiency history, peripheral vessel disease history, CABG history, revascularization History, creatinine clearance, left ventricular fraction and SYNTAX score are different (all P < 0.05) (Table 1).

**Table 1:** Characteristics of study population by treatment strategies

Variables	PCI (N = 1305)	CABG (N = 899)	MT (N = 913)	P value
Age (Years)	60.5 ± 10.1	61.3 ± 8.4	63.1 ± 9.8	< 0.001
Body mass index (kg/m^2^)	26.1 ± 2.9	25.9 ± 2.9	25.8 ± 3.1	0.163
Male	977 (74.9)	696 (77.4)	660 (72.3)	0.042
Hypertension history	943 (72.3)	632 (70.3)	643 (70.4)	0.514
Hyperlipidemia history	791 (60.6)	509 (56.6)	546 (59.8)	0.158
Chronic kidney disease history	7 (0.5)	15 (1.7)	19 (2.1)	0.004
COPD history	13 (1.0)	10 (1.1)	16 (1.8)	0.261
PAD history	64 (4.9)	121 (13.5)	106 (11.6)	< 0.001
Smoking history	673 (51.6)	449 (49.9)	474 (51.9)	0.426
PCI history	180 (13.8)	88 (9.8)	126 (13.8)	0.842
CABG history	49 (3.8)	1 (0.1)	55 (6.0)	0.049
Revascularization history	307 (46.2)	130 (19.6)	227 (34.2)	< 0.001
hsCRP (mg/L)	3.9 ± 6.1	3.8 ± 5.4	4.2 ± 4.8	0.455
Creatinine clearance (mL/min)	89.0 ± 28.5	86.8 ± 26.3	83.0 ± 28.2	< 0.001
LVEF	59.2 ± 8.8	57.8 ± 9.5	54.9 ± 11.7	< 0.001
SYNTAX score	22.2 ± 9.3	30.6 ± 9.2	26.3 ± 12.9	< 0.001

Values are mean ± SD or n (%)

*PCI* percutaneous coronary intervention, *CABG* coronary artery bypass grafting, *MT* medical treatment, *COPD* chronic obstructive pulmonary diseases, *PVD* peripheral vessel disease, *hsCRP* high-sensitivity C-reactive protein, *LVEF* left ventricular ejection fraction, *SYNTAX* Synergy between Percutaneous Coronary Intervention with Taxus and Cardiac Surgery

### Outcome according to the three therapeutic strategies

**Fig. 2:**
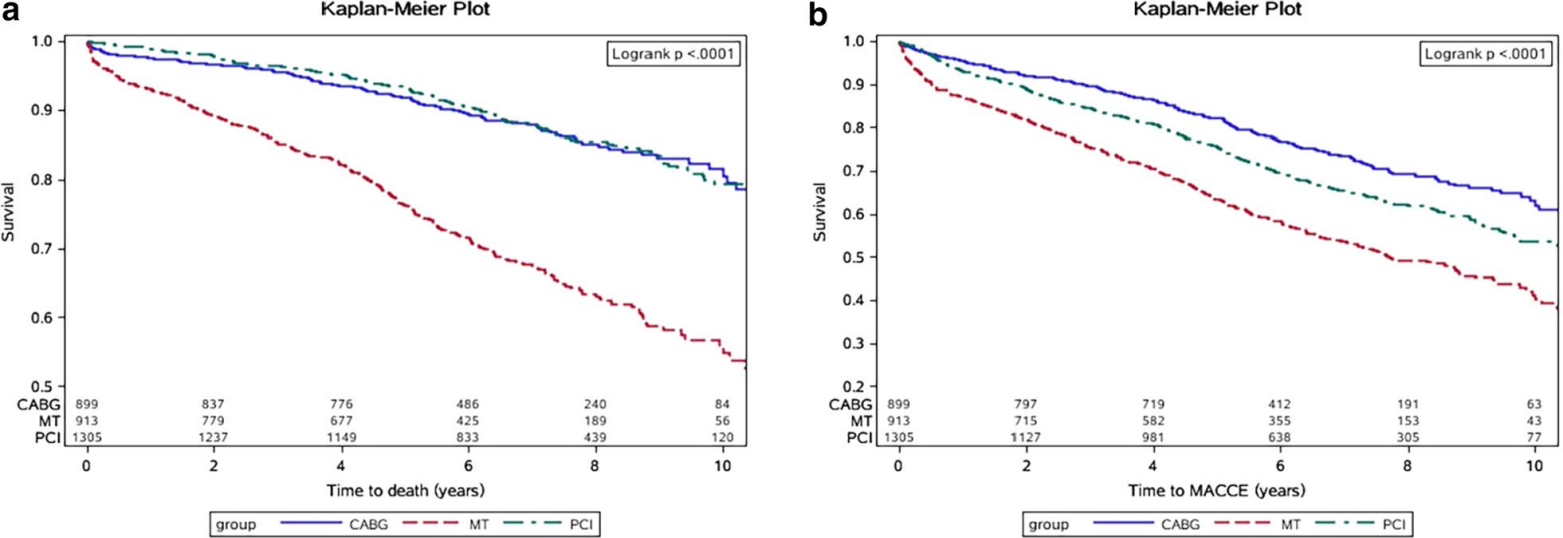
Kaplan–Meier survival curve analysis of death (**a**) (log-rank p < 0.0001) and MACCE (**b**) (log-rank p < 0.0001) according to different strategies Including PCI, CABG and MT treatment. *PCI* percutaneous coronary intervention, *CABG* coronary artery bypass grafting, *MT*  medical treatment, *MACCE*  major adverse cardiac and cerebrovascular events

Mean follow-up was for 6.3 ± 2.6 years, there were 573 (18.4%) deaths, and 1094 (35.1%) MACCE occurred. Kaplan–Meier curve analysis showed that the 10-year cumulative mortality rates in the PCI, CABG, and MT groups were 16.7%, 17.1%, and 41.5%, respectively (log-rank, P < 0.0001) (Fig. 2).

After Cox multi-factor adjustment, all-cause death in the PCI group (HR 0.40, 95% CI 0.32–0.51, P < 0.001) and CABG group (HR 0.33, 95% CI 0.26–0.44, P < 0.001) was significantly lower than that in the MT group. However, there was no significant difference in the risk of death between the CABG and PCI groups (HR 0.83, 95% CI 0.62–1.12, P = 0.222) (Table 2). The risk of MACCE in the PCI group (HR 0.71, 95% CI 0.60–0.84, P < 0.001) and the CABG group (HR 0.48, 95% CI 0.39–0.57, P < 0.001) was significantly lower than that in the MT group, while the CABG group had a lower risk of MACCE compared with the PCI group (HR 0.67, 95% CI 0.55–0.81, P < 0.001) (Table 2). Further analysis showed that the benefit of the reduction in MACCE was mainly due to CABG reducing the risk of MI (HR 0.22, 95% CI 0.12–0.41, P < 0.001), but at the same time increasing the risk of stroke (HR 1.51, 95% CI 1.08–2.11, P = 0.016).

**Table 2:** Hazard ratios for death and MACCE associated with treatment strategies

	MT group (N = 913)		PCI group (N = 1305)		CABG group (N = 899)	
	HR (95% CI)	P value	HR (95% CI)	P value	HR (95% CI)	P value
Death						
Crude Model	1.00 (ref)	–	0.33 (0.27, 0.40)	< 0.0001	0.34 (0.27, 0.42)	< 0.0001
Multivariate adjusted Model	1.00 (ref)	–	0.40 (0.32, 0.51)	< 0.0001	0.33 (0.26, 0.44)	< 0.0001
Crude Model	3.03 (2.51, 3.66)	< 0.0001	1.00 (ref)	–	1.03 (0.81, 1.30)	0.8396
Multivariate adjusted Model	2.50 (1.98, 3.16)	< 0.0001	1.00 (ref)	–	0.83 (0.62, 1.12)	0.2215
MACCE						
Crude Model	1.00 (ref)		0.66 (0.58, 0.75)	< 0.0001	0.48 (0.41, 0.57)	< 0.0001
Multivariate adjusted Model	1.00 (ref)		0.71 (0.60, 0.84)	< 0.0001	0.48 (0.39, 0.57)	< 0.0001
Crude Model	1.52 (1.33, 1.73)	< 0.0001	1.00 (ref)	–	0.73 (0.62, 0.86)	0.0001
Multivariate adjusted Model	1.41 (1.20, 1.66)	< 0.0001	1.00 (ref)	–	0.67 (0.55, 0.81)	< 0.0001

*MACCE* major adverse cardiac and cerebrovascular events, *HR* hazard ratios, *MT* medical treatment, *PCI* percutaneous coronary intervention, *CABG* coronary artery bypass grafting

### Effect of the SYNTAX score

When all-cause death was the clinical endpoint, regardless of the SYNTAX subgroups, patients in the PCI and CABG groups showed a significantly lower risk than those in the MT group (all P < 0.05). However, regardless of the SYNTAX subgroup, there was no difference in the risk of all-cause death between the CABG and PCI groups (all P > 0.05) (Table 3).

**Table 3:** Effect of SYNTAX score grouping on death and MACCE outcomes of different treatment strategies

	MT group (N = 913)		PCI group (N = 1305)		CABG group (N = 899)	
	HR (95% CI)	P value	HR (95% CI)	P value	HR (95% CI)	P value
Death						
SYNTAX scores ≤ 22	1.00 (ref)	–	0.44 (0.29, 0.66)	0.0001	0.40 (0.19, 0.84)	0.0148
SYNTAX scores 22–32	1.00 (ref)	–	0.31 (0.21, 0.46)	< 0.0001	0.27 (0.18, 0.42)	< 0.0001
SYNTAX scores ≥ 33	1.00 (ref)	–	0.48 (0.29, 0.80)	0.0043	0.38 (0.25, 0.57)	< 0.0001
SYNTAX scores ≤ 22	2.29 (1.51, 3.47)	0.0001	1.00 (ref)	–	0.92 (0.45, 1.90)	0.8256
SYNTAX scores 22–32	3.20 (2.18, 4.70)	< 0.0001	1.00 (ref)	–	0.87 (0.54, 1.41)	0.5641
SYNTAX scores ≥ 33	2.09 (1.26, 3.45)	0.0043	1.00 (ref)	–	0.79 (0.46, 1.36)	0.3976
MACCE						
SYNTAX scores ≤ 22	1.00 (ref)	–	0.92 (0.70, 1.22)	0.5589	0.55 (0.34, 0.88)	0.0126
SYNTAX scores 22–32	1.00 (ref)	–	0.63 (0.48, 0.83)	0.0009	0.45 (0.33, 0.61)	< 0.0001
SYNTAX scores ≥ 33	1.00 (ref)	–	0.57 (0.39, 0.84)	0.0038	0.47 (0.34, 0.64)	< 0.0001
SYNTAX scores ≤ 22	1.09 (0.82, 1.44)	0.5589	1.00 (ref)	–	0.59 (0.38, 0.93)	0.0215
SYNTAX scores 22–32	1.59 (1.21, 2.10)	0.0009	1.00 (ref)	–	0.71 (0.52, 0.97)	0.0299
SYNTAX scores ≥ 33	1.75 (1.20, 2.56)	0.0038	1.00 (ref)	–	0.82 (0.55, 1.21)	0.3181

*SYNTAX* Synergy between Percutaneous Coronary Intervention with Taxus and Cardiac Surgery, *MACCE* major adverse cardiac and cerebrovascular events, *MI* myocardial infarction, *MT* medical treatment, *PCI* percutaneous coronary intervention, *CABG* coronary artery bypass grafting

When MACCE was the clinical endpoint, PCI failed to reduce the risk of MACCE compared with MT (P = 0.559) in patients with a SYNTAX score of ≤ 22. However, CABG was associated with a lower risk compared with MT (HR 0.55, 95% CI 0.34–0.88) and PCI (HR 0.59, 95% CI 0.38–0.93). In patients with a SYNTAX score of 23–32, PCI (HR 0.63, 95% CI 0.48–0.83) and CABG (HR 0.45, 95% CI 0.33–0.61) were associated with a lower risk of MACCE compared with MT. CABG was also associated with a lower risk of MACCE compared with PCI (HR 0.71, 95% CI 0.52–0.97). In patients with a SYNTAX score of ≥ 33, PCI (HR 0.57, 95% CI 0.39–0.84) and CABG (HR 0.47, 95% CI 0.34–0.64) were still associated with a lower risk of MACCE compared with MT. However, CABG failed to lower the risk of the MACCE endpoint compared with PCI (P = 0.318) (Table 3).

Regardless of the SYNTAX group, CABG significantly reduced the risk of MI compared with PCI (P < 0.05). For the risk of stroke, there was no difference between CABG and PCI when the SYNTAX score was ≤ 22 (P = 0.946). However, in the medium and high score SYNTAX groups, CABG had a higher risk of stroke than PCI (both P < 0.05) (Table 4).

**Table 4:** Effect of SYNTAX score grouping on MI and stroke outcomes of different treatment strategies

	MT group (N = 913)		PCI group (N = 1305)		CABG group (N = 899)	
	HR (95% CI)	P value	HR (95% CI)	P value	HR (95% CI)	P value
MI						
SYNTAX scores ≤ 22	0.77 (0.41, 1.48)	0.4372	1.00 (ref)	–	0.11 (0.01, 0.78)	0.0278
SYNTAX scores 22–32	0.57 (0.26, 1.24)	0.1589	1.00 (ref)	–	0.15 (0.05, 0.44)	0.0005
SYNTAX scores ≥ 33	0.56 (0.22, 1.40)	0.2142	1.00 (ref)	––	0.23 (0.08, 0.65)	0.0057
Stroke						
SYNTAX scores ≤ 22	0.91 (0.51, 1.65)	0.7610	1.00 (ref)	–	1.02 (0.51, 2.07)	0.9463
SYNTAX scores 22–32	0.77 (0.36, 1.63)	0.4908	1.00 (ref)	–	1.87 (1.10, 3.18)	0.0214
SYNTAX scores ≥ 33	1.88 (0.73, 4.84)	0.1888	1.00 (ref)	–	2.39 (1.02, 5.60)	0.0447

*SYNTAX* Synergy between Percutaneous Coronary Intervention with Taxus and Cardiac Surgery, *MI* myocardial infarction, *MT* medical treatment, *PCI* percutaneous coronary intervention, *CABG* coronary artery bypass grafting

### Subgroup analysis between PCI and CABG for all-cause death

**Fig. 3:**
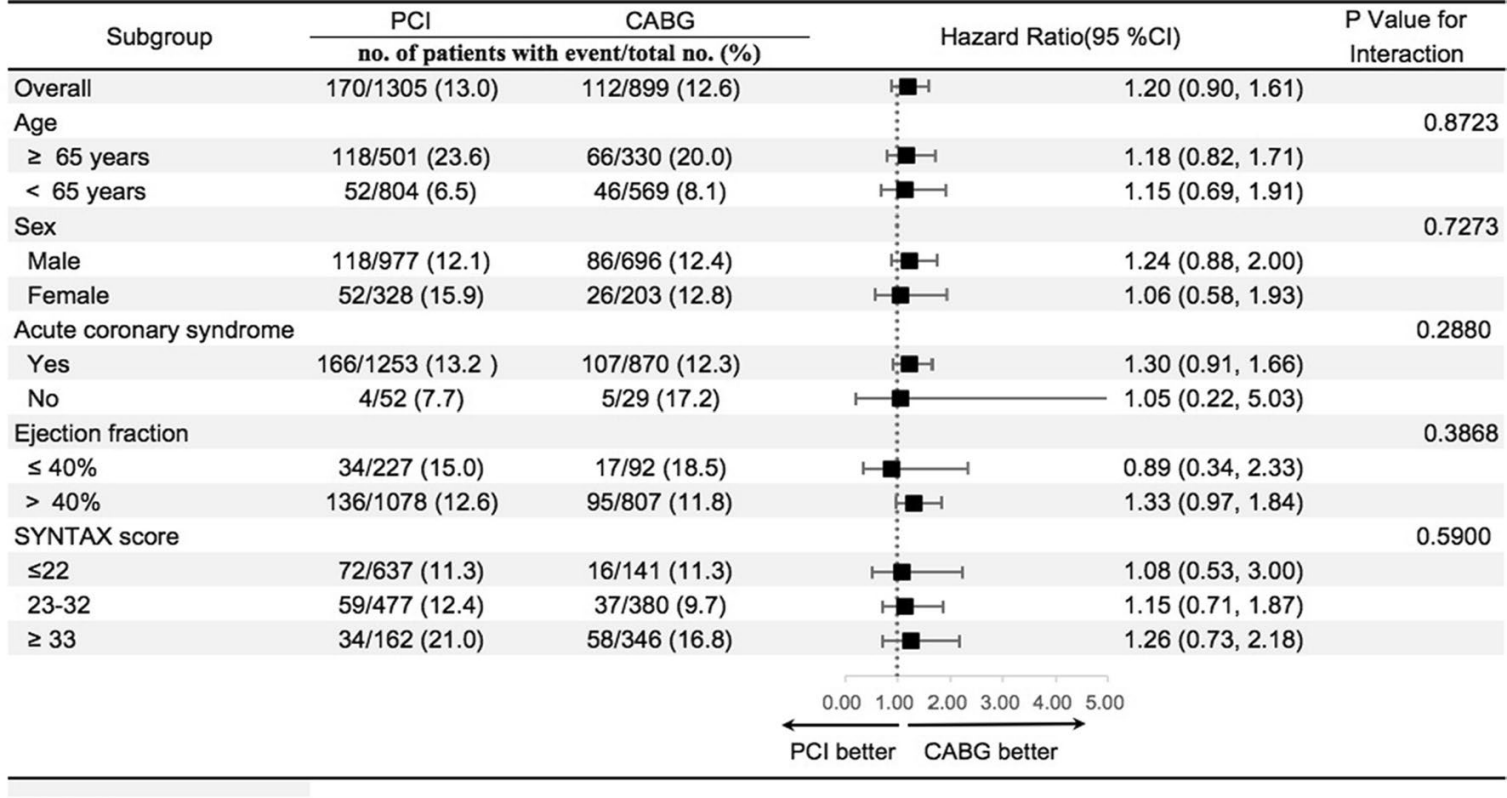
Subgroup Analyses of Primary End Point. Hazard ratios and 95% confidence intervals are shown for the primary composite end point of all-cause death. P value represents interaction test between the variable and the relative treatment effect. *PCI* percutaneous coronary intervention, *CABG* coronary artery bypass grafting, *SYNTAX* Synergy between Percutaneous Coronary Intervention with Taxus and Cardiac Surgery

There was no difference in all-cause death in patients with diabetes between the PCI and CABG groups. This result was consistent in different subgroups, regardless of age, sex, acute coronary syndrome, left ventricular fraction, and the SYNTAX score (Fig. 3).

## Discussion

In this large-sample and prospective cohort study in the real world, the main results were as follows. In patients with diabetes and TVD, PCI and CABG treatment were associated with a lower risk of all-cause death and MACCE than MT alone. CABG was not associated with a lower risk of death, but was associated with a lower risk of MACCE compared with PCI. This was mainly achieved by reducing the risk of MI, but increasing the risk of stroke.

### Revascularization was associated with lower risk compare with MT alone

Our study showed that in patients with diabetes and TVD, PCI and CABG was associated with a lower risk of all-cause death and MACCE during follow-up, which suggested the importance of revascularization for these patients. Previous studies have shown that patients with diabetes have a worse prognosis [16] and benefits more from revascularization than non-diabetic patients [17]. However, it should be pointed out that patients in the MT group may have had different conditions, including a coronary artery with simple lesions (low SYNTAX scores) in which revascularization was not required; additionally, another condition is where a coronary artery with complex lesions (high SYNTAX scores) could not have revascularization performed. Therefore, we conducted further analysis of the subgroup of SYNTAX scores. We found that PCI and CABG were better than MT, except for when the SYNTAX score was ≤ 22 and the risk of MACCE was not associated with a lower risk in the PCI group compared with the MT group. The BARI-2D study [18] included 1550 patients with diabetes and stable coronary heart disease (CHD) showed that, in the group with a low SYNTAX score (≤ 22), there was no significant difference in events (death, MI, or stroke) between the PCI and MT groups, which is consistent with our results. In the future, for these patients with diabetes and a low SYNTAX score, further benefits may be obtained if PCI is guided by functional tests, such as fractional flow reserve. However, the BARI-2D study also showed that in the low SYNTAX score group, CABG was not superior to MT. This finding may be related to the fact that patients enrolled in the BRRI-2D study had stable CHD and non-TVD disease, and therefore, the event risk was relatively low.

### Outcome of all-cause death after CABG and PCI

At present, most studies on patients with diabetes and MVD use MACCE as the clinical endpoint. Few studies use death as the clinical endpoint, and notably, the results are inconsistent. We conducted a 6.3 years follow-up in the real world and found that after multi-factor adjustment, there was no difference in the risk of all-cause death in patients with diabetes and TVD between CABG and PCI. To avoid interference of the complexity of lesions on the results, we added the baseline SYNTAX score to multivariate Cox analysis and grouped patients according to the SYNTAX score, but the results remained unaltered. In the FREEDOM study, a higher mortality rate was observed in the PCI group than in the CABG group at 3.8 years of follow-up (P = 0.049) [3]. However, this difference disappeared when the follow-up was extended to 7.5 years (943 patients) (P = 0.076) [7]. A large meta-analysis [6] included patients with multivessel (TVD 58.6%) or left main coronary artery disease who did not present with acute myocardial infarction, especially those with high-complexity lesions and diabetes, CABG reduced the risk of death compared with PCI (new-generation drug stents 34.2%). In a real-world study [19, 20], among diabetic patients with multivessel and/or left main disease, after 7 months, surgical revascularization was associated with a significant survival benefit. However, Bangalore et al. [9] found that patients with everolimus drug-eluting stents had a similar risk of death compared with CABG in a long-term follow-up study of patients with diabetes and MVD. The 10-year results of the newly published SYNTAX study [10] also showed that there was no difference in the risk of death between CABG and PCI in patients with diabetes, but CABG reduced the risk of death in patients with TVD compared with PCI. In the study by Naito et al. [21], even in the patients ≥ 65 years old, DM, and MVD, the all-cause mortality rate was not significantly different between the CABG and PCI with DES. The results of these studies are inconsistent, which may be related to the following factors: (1) different follow-up times and sample sizes; (2) some studies were only selected for stable CHD; and (3) whether the SYNTAX score and other influence factors were included in the multivariate analysis. Our study included 3117 patients with diabetes and TVD, who were followed up for 6.3 years, and we conducted subgroup analysis. The results were consistent in different subgroups, regardless of age, sex, acute coronary syndrome, left ventricular fraction, and the SYNTAX score.

The reason why there was no difference in the risk of death between CABG and PCI in patients with diabetes and TVD is not completely clear yet, but the reasons might be as follows. Diabetic patients tend to have more diffuse lesions, involving small vessels and micro-vascular, and higher plaque burden, which is different from non-diabetic patients. The revascularization of PCI and CABG can only solve the stenosis of main epicardial coronary arteries, but could not improve the lesions of small vessels and micro-vascular. As a result, the outcomes of revascularization in diabetic patients may be different from those in non-diabetic patients in previous study [22]. It is generally believed that SYNTAX score will affect the choice of treatment strategies for patients with TVD and diabetes [23]. However, interestingly, our study did not see the effect of SYNATX scores on all-cause deaths with different revascularization. We consider that this may be related to the fact that the SYNTAX score mainly reflects the degree of stenosis of the main epicardial coronary artery, but does not reflect the degree of lesion of small vessels and micro-vascular which play an important role in coronary circulation [24]. However, patients with diabetes often have diffuse small vessels and micro-vascular lesions, which may affect the prognosis. In addition, although CABG reduces the risk of MI compared with PCI, there is still a risk of cerebrovascular events, renal insufficiency, tumors [25], and other fatal comorbidities in patients with diabetes. This suggests that we should not only pay attention to coronary artery stenosis and vulnerable plaques, but also pay attention to the high-risk patients [26]. In clinical work, in patients with diabetes and TVD, attention should also be paid to comprehensive strategies of these patients, such as improvement of lifestyle, lipoprotein-lowering therapy, antithrombotic therapy, sodium-dependent glucose transporters 2 inhibitors, Glucagon-like peptide-1 [27], and possible anti-inflammatory therapy [28]. This may further reduce the risk of death [29].

### Outcome of MACCE between CABG and PCI

Our intermediate-term follow-up of patients with diabetes and TVD in the real world showed that CABG was associated with a lower risk of MACCE and MI, but also a higher risk of stroke, compared with PCI. Our results are consistent with those of most previous studies as follows. In the FREEDOM trial [3], which included 1900 patients with CHD complicated by diabetes, CABG significantly reduced MACCE (all-cause death, MI, or non-fatal stroke) compared with PCI during the 3.8-year follow-up. The SYNTAX study [4] included patients with TVD and/or left main coronary artery disease. During the 5-year follow-up in this study, CABG reduced MACCE endpoints compared with PCI. Further analysis showed that CABG reduced MI and revascularization compared with PCI, but there was no difference in the risk of stroke and all-cause death, and no interaction was found in diabetic subgroups. A meta-analysis [5] included four randomized controlled studies, including 3052 patients with diabetes and MVD, which showed that CABG had a lower risk of MACCE (composite of death, MI, and stroke) than PCI. In a real-world study [30], CABG was associated with significantly lower rates of death, MI and target vessel revascularization in patients with multivessel disease or left main coronary artery, particularly for patients with diabetes. At present, relatively consistent studies show that CABG reduces MACCE compared with PCI, CABG is still important in patients with diabetes and TVD, this should be considered when choosing treatment strategies for patients, and it is also in line with the current guidelines [11]. It should be noted, the new generation of drug stents has thinner struts, and better biocompatible polymer coating and newer antiproliferative agents have reduced the rates of cardiovascular events in DM patients with PCI [31]. Recently, the everolimus-eluting bioresorbable scaffolds treatment in DM patients showed comparable midterm safety and efficacy outcomes when historically compared with modern DES [32]. In the future, with the development of PCI technology and the renewal of stents, perhaps the gap affected by PCI and CABG in the prognosis of patients with diabetes will be further narrowed.

Our results suggest that the benefit of a reduction in risk of MACCE in patients with CABG compared with those with PCI is mainly due to a reduction in the risk of MI (HR 0.22). Additionally, this benefit was observed in different subgroups of the SYNTAX score. The main reason for this finding may be that patients with CABG received more complete revascularization than those with PCI, and the bridging vessels covered more coronary artery lesions. These results are consistent with those of previous studies [3, 4, 33]. However, at the same time, CABG increased the risk of stroke compared with PCI. Further subgroup analysis showed that the increase in risk of stroke mainly occurred in the medium and high SYNTAX score groups (23–32 and ≥ 33), but there was no such difference in the low SYNTAX score group (≤ 22). Previous studies and meta-analyses have also shown similar results [7, 8, 34]. However, the specific mechanism of an increased risk of stroke is not clear. This mechanism may be related to a difference in drug use or an increase in atrial fibrillation after CABG [35]. Our study suggests that for patients with diabetes mellitus and TVD who are ready for CABG, the carotid artery and ascending aorta should be carefully evaluated before the operation, and appropriate antithrombotic therapy should be provided to reduce the risk of stroke.

Our study provides reliable real-world data and intermediate-term follow-up results of different treatment strategies for MT, PCI and CABG. The results suggest the importance of appropriate revascularization for diabetic patients with TVD. Moreover, in the future, in order to reduce the all-cause mortality of diabetic patients with TVD, we perhaps should strengthen comprehensive treatment in addition to PCI or CABG. The benefit / risk ratio of different revascularization methods should be discussed by the heart team in patients with multi-vessel disease of diabetes, especially for those patients with complex lesions or concomitant diseases [36, 37]. In addition, attention should be paid to combining the patients’ own opinions.

### Limitations

The present study has some inevitable limitations. First, our study was a single-center, observational study, which may have limited the generalizability of its findings. Second, although we attempted to control for multiple variables, our study was inevitably affected by some confounding factors. Third, there was a lack of specific information in the database, such as exercise treadmill, fractional flow reserve and coronary artery bridges. In the future, more randomized, controlled trials are required to validate our results.

## Conclusions

In this real-world study, after a mean follow-up of 6.3 ± 2.6 years of patients with diabetes and TVD, PCI and CABG are associated with a lower risk of death and MACCE compared with MT alone. We do not observe the significant difference in the risk of all-cause death between CABG and PCI, but CABG is indeed associated with a lower risk of MACCE compared with PCI. This is due to the fact that CABG is significantly associated with a lower risk of MI compared with PCI, but also a higher risk of stroke.

## Data Availability

Due to ethical restrictions related to the consent given by subjects at the time of study commencement, our datasets are available from the corresponding author upon reasonable request after permission of the Institutional Review Board of State Key Laboratory of Cardiovascular Disease, Fuwai Hospital, National Center for Cardiovascular Diseases.

## Abbreviations

MVD: Multi-vessel coronary artery disease
PCI: Percutaneous coronary intervention
CABG: Coronary artery bypass grafting
TVD: Triple-vessel coronary disease
MACCE: Major adverse cardiovascular and cerebrovascular events
MI: Myocardial infarction
FREEDOM: Future revascularization evaluation in patients with diabetes mellitus: optimal management of multivessel disease
SYNTAX: Synergy between percutaneous coronary intervention with taxus and cardiac surgery
MT: Medical treatment
CHD: Coronary heart disease
HR: Hazard ratio
CI: Confidence interval

## References

[1] Donahoe SM, Stewart GC, McCabe CH, Mohanavelu S, Murphy SA, Cannon CP, Antman EM (2007). Diabetes and mortality following acute coronary syndromes. JAMA.

[2] Rawshani A, Franzén S, Eliasson B, Svensson AM, Miftaraj M, McGuire DK (2017). Mortality and cardiovascular disease in type 1 and type 2 diabetes. N Engl J Med.

[3] Farkouh ME, Domanski M, Sleeper LA, Siami FS, Dangas G, Mack M (2012). Strategies for multivessel revascularization in patients with diabetes. N Engl J Med.

[4] Mohr FW, Morice MC, Kappetein AP, Feldman TE, Ståhle E, Colombo A (2013). Coronary artery bypass graft surgery versus percutaneous coronary intervention in patients with three-vessel disease and left main coronary disease: 5-year follow-up of the randomised, clinical SYNTAX trial. Lancet.

[5] Hakeem A, Garg N, Bhatti  S, Rajpurohit N, Ahmed Z, Uretsky BF (2013). Effectiveness of percutaneous coronary intervention with drug-eluting stents compared with bypass surgery in diabetics with multivessel coronary disease: comprehensive systematic review and meta-analysis of randomized clinical data. Journal of the American Heart Association.

[6] Head SJ, Milojevic M, Daemen J, Ahn JM, Boersma E, Christiansen EH (2018). Mortality after coronary artery bypass grafting versus percutaneous coronary intervention with stenting for coronary artery disease: a pooled analysis of individual patient data. Lancet.

[7] Farkouh ME, Domanski M, Dangas GD, Godoy LC, Mack MJMJ, Siami FS (2019). Long-term survival following multivessel revascularization in patients with diabetes: the FREEDOM follow-on study. J Am Coll Cardiol.

[8] Verma S, Farkouh ME, Yanagawa B, Fitchett DH, Ahsan MR, Ruel M (2013). Comparison of coronary artery bypass surgery and percutaneous coronary intervention in patients with diabetes: a meta-analysis of randomised controlled trials. Lancet Diabetes Endocrinol.

[9] Bangalore S, Guo Y, Samadashvili Z, Blecker S, Xu J, Hannan EL (2015). Everolimus eluting stents versus coronary artery bypass graft surgery for patients with diabetes mellitus and multivessel disease. Circ Cardiovasc Intervent.

[10] Thuijs DJ, Kappetein AP, Serruys PW, Mohr FW, Morice MC, Mack MJ (2019). Percutaneous coronary intervention versus coronary artery bypass grafting in patients with three-vessel or left main coronary artery disease: 10-year follow-up of the multicentre randomised controlled SYNTAX trial. Lancet..

[11] Neumann FJ, Sousa-Uva M, Ahlsson A, Alfonso F, Banning AP, Benedetto U (2019). 2018 ESC/EACTS Guidelines on myocardial revascularization. Eur Heart J.

[12] Zhao X, Jiang L, Xu L, Tian J, Xu Y, Zhao Y, Feng X, Wu Y, Zhang Y, Wang D (2019). Predictive value of in-hospital white blood cell count in Chinese patients with triple-vessel coronary disease. Eur J Prevent Cardiol.

[13] Serruys PW, Morice MC, Kappetein AP, Colombo A, Holmes DR, Mack MJ, Stahle E, Feldman TE, van den Brand M, Bass EJ (2009). Percutaneous coronary intervention versus coronary-artery bypass grafting for severe coronary artery disease. N Engl J Med.

[14] Task Force M, Montalescot G, Sechtem U, Achenbach S, Andreotti F, Arden C, Budaj A, Bugiardini R, Crea F, Cuisset T (2013). 2013 ESC guidelines on the management of stable coronary artery disease: the Task Force on the management of stable coronary artery disease of the European Society of Cardiology. Eur Heart J.

[15] Jneid H, Anderson JL, Wright RS, Adams CD, Bridges CR, Casey DE, Ettinger SM, Fesmire FM, Ganiats TG, Lincoff AM (2012). 2012 ACCF/AHA focused update of the guideline for the management of patients with unstable angina/non-ST-elevation myocardial infarction (updating the 2007 guideline and replacing the 2011 focused update): a report of the American College of Cardiology Foundation/American Heart Association Task Force on Practice Guidelines. J Am Coll Cardiol.

[16] Ram E, Sternik L, Klempfner R, Iakobishvili Z, Fisman E, Tenenbaum A, Zuroff E, Peled Y, Raanani E (2020). Type 2 diabetes mellitus increases the mortality risk after acute coronary syndrome treated with coronary artery bypass surgery. Cardiovasc Diabetol.

[17] Tsai C, Huang W, Teng H, Tsai Y, Lu T (2020). Long term clinical impact of successful recanalization of chronic total occlusion in patients with and without type 2 diabetes mellitus. Cardiovasc Diabetol.

[18] Ikeno F, Brooks MM, Nakagawa K, Kim MK, Kaneda H, Mitsutake Y (2017). SYNTAX score and long-term outcomes: the BARI-2D trial. J Am Coll Cardiol.

[19] Ram E, Goldenberg I, Kassif Y, Segev A, Lavee J, Einhorn-Cohen M, Raanani E (2018). Real-life characteristics and outcomes of patients who undergo percutaneous coronary intervention versus coronary artery bypass grafting for left main coronary artery disease: data from the prospective Multi-vessel Coronary Artery Disease (MULTICAD) Israeli Registry. Eur J Cardio Thorac Surg.

[20] Ram E, Goldenberg I, Sternik L, Peled Y, Segev A, Kogan A, Vorobeichik Pechersky D, Shlomo N, Raanani E (2019). Real-world referral pattern and outcomes of diabetic patients who undergo revascularization: data from the prospective Multi-vessel Coronary Artery Disease (MULTICAD) Israeli Registry†. Eur J Cardio Thorac Surg.

[21] Naito R, Miyauchi K, Konishi H, Tsuboi S, Ogita M, Dohi T, Kajimoto K, Kasai T, Tamura H, Okazaki S (2016). Comparing mortality between coronary artery bypass grafting and percutaneous coronary intervention with drug-eluting stents in elderly with diabetes and multivessel coronary disease. Heart Vessels.

[22] Cui K, Lyu S, Liu H, Song X, Yuan F, Xu F, Zhang M, Wang W, Zhang M, Zhang D (2019). Staged complete revascularization or culprit-only percutaneous coronary intervention for multivessel coronary artery disease in patients with ST-segment elevation myocardial infarction and diabetes. Cardiovasc Diabetol.

[23] Stanetic B, Ostojic M, Kovacevic-Preradovic T, Kos L, Stanetić K, Nikolic A, Bojic M, Huber K (2020). ApPropRiateness of myOcardial revascUlarization assessed by SYNTAX Scores in patients with type 2 diabetes melliTus: the PROUST study. Postepy w kardiologii interwencyjnej = Adv Intervent Cardiol.

[24] Herrmann J, Kaski J, Lerman A (2012). Coronary microvascular dysfunction in the clinical setting: from mystery to reality. Eur Heart J.

[25] Wu D, Hu D, Chen H, Shi G, Fetahu IS, Wu F (2018). Glucose-regulated phosphorylation of TET2 by AMPK reveals a pathway linking diabetes to cancer. Nature..

[26] Arbab-Zadeh A, Fuster V (2019). From detecting the vulnerable plaque to managing the vulnerable patient: JACC State-of-the-Art review. J Am Coll Cardiol.

[27] Das SR, Everett BM, Birtcher KK, Brown JM, Cefalu WT, Januzzi JL (2018). 2018 ACC expert consensus decision pathway on novel therapies for cardiovascular risk reduction in patients with type 2 diabetes and atherosclerotic cardiovascular disease: a report of the American College of Cardiology Task Force on Expert Consensus Decision Pathways. J Am Coll Cardiol..

[28] Ridker PM, Everett BM, Thuren T, MacFadyen JG, Chang WH, Ballantyne C, Fonseca F, Nicolau J, Koenig W, Anker SD (2017). Antiinflammatory therapy with canakinumab for atherosclerotic disease. N Engl J Med.

[29] Patel KV, Pandey A, de Lemos JA (2018). Conceptual framework for addressing residual atherosclerotic cardiovascular disease risk in the era of precision medicine. Circulation..

[30] Fortuna D, Nicolini F, Guastaroba P, De Palma R, Di Bartolomeo S, Saia F, Pacini D, Grilli R (2013). Coronary artery bypass grafting vs percutaneous coronary intervention in a ‘real-world’ setting: a comparative effectiveness study based on propensity score-matched cohorts. Eur J Cardio Thorac Surg.

[31] Guandalini G, Bangalore S (2018). The potential effects of new stent platforms for coronary revascularization in patients with diabetes. Can J Cardiol.

[32] Hommels T, Hermanides R, Rasoul S, Berta B, IJsselmuiden A, Jessurun G, Benit E, Pereira B, De Luca G, Kedhi E (2019). Everolimus-eluting bioresorbable scaffolds for treatment of coronary artery disease in patients with diabetes mellitus: the midterm follow-up of the prospective ABSORB DM Benelux study. Cardiovasc Diabetol.

[33] Yan Y, Zhang M, Yuan F, Liu H, Wu D, Fan Y, Guo X, Xu F, Zhang M, Zhao Q (2019). Successful revascularization versus medical therapy in diabetic patients with stable right coronary artery chronic total occlusion: a retrospective cohort study. Cardiovasc Diabetol.

[34] Head SJ, Milojevic M, Daemen J, Ahn JM, Boersma E, Christiansen EH (2018). Stroke rates following surgical versus percutaneous coronary revascularization. J Am Coll Cardiol.

[35] Echahidi N, Pibarot P, O’Hara G, Mathieu P (2008). Mechanisms, prevention, and treatment of atrial fibrillation after cardiac surgery. J Am Coll Cardiol..

[36] Ram E, Goldenberg I, Kassif Y, Segev A, Lavee J, Shlomo N, Raanani E (2018). Comparison of patients with multivessel disease treated at centers with and without on-site cardiac surgery. J Thorac Cardiovasc Surg.

[37] Ram E, Raanani E, Klempfner R, Peled Y, Sternik L, Segev A (2020). Midterm outcomes of patients with multivessel disease treated at centers with and without on-site cardiac surgery services. J Thorac Cardiovasc Surg.

